# Church-based problem-solving therapy for adolescent girls and young women with a history of gender-based violence in Zambia: study protocol for a hybrid type 1 randomized controlled trial

**DOI:** 10.1186/s13063-026-09625-3

**Published:** 2026-04-10

**Authors:** Charisse V. Ahmed, Moomba Thornicroft, Katongo Chileshe, Bristol M. Ntebeka, Kathryn Dovel, Laura M. Bogart, Noé Rubén Chávez, Francisca Tshitenge Bwalya, Merrian J. Brooks, Dallas Swendeman

**Affiliations:** 1 Department of Family Health Care Nursing, San Francisco (UCSF) School of Nursing, University of California, San Francisco, CA USA; 2 A Place Called Home-Ikhaya, Lusaka, Zambia; 3 Department of Gender Studies, School of Humanities and Social Sciences, University of Zambia, Lusaka, Zambia; 4 Department of Epidemiology and Biostatistics, University of Zambia, Lusaka, Zambia; 5 Los Angeles (UCLA) David Geffen School of Medicine, Division of Infectious Diseases, University of California, California, USA; 6 Department of Psychiatry, RAND, Santa Monica, CA and Charles R. Drew University of Medicine and Science, Los Angeles, CA USA; 7 Department of Social Sciences and Humanities, Charles R. Drew University of Medicine and Science, Los Angeles, USA; 8 Department of Clinical Care, Chainama Hills Hospital, Lusaka, Zambia; 9 University of Pennsylvania, Perelman School of Medicine, Philadelphia, PA USA; 10 Semel Institute for Neuroscience & Human Behavior, University of California, Los Angeles (UCLA), Los Angeles, CA USA; 11 Department of Epidemiology, UCLA Fielding School of Public Health, Los Angeles, CA USA

**Keywords:** Gender-based violence (GBV), Adolescent girls and young women (AGYW), Problem-solving therapy (PST), Church-based intervention, Hybrid type 1 implementation-effectiveness trial, HIV prevention and treatment, Mental health, Zambia, Lay counselors, Implementation science

## Abstract

**Background:**

Adolescent girls and young women (AGYW) in sub-Saharan Africa face a disproportionate burden of gender-based violence (GBV), HIV risk, and mental health challenges. Despite the demonstrated effectiveness of psychological interventions such as problem-solving therapy (PST), there is limited evidence on culturally adapted models for GBV-exposed AGYW that concurrently address HIV and mental health, particularly in settings with constrained mental health infrastructure.

**Objective:**

This study outlines the protocol for a randomized controlled trial evaluating *Mpata Yathu*, a church-based adaptation of the Friendship Bench intervention, which delivers lay counselor-led PST to AGYW with a history of GBV in Lusaka, Zambia.

**Methods:**

A hybrid implementation-effectiveness type 1 randomized controlled trial (intervention and waitlist control arms) will be conducted among 90 AGYW, aged 15 to 24, per arm with moderate depressive symptoms, lifetime GBV exposure, and HIV risk or diagnosis. The intervention comprises six individual PST sessions delivered over 3 months in church spaces. Implementation outcomes (feasibility, acceptability, fidelity), common mental disorder (CMD) symptoms (primary outcome), and exploratory outcomes (depression, anxiety, PTSD, HIV engagement, GBV attitudes, childhood adversity, coping self-efficacy) will be assessed at baseline, 3, and 6 months. The trial is powered to detect changes in CMD symptoms only. Mixed-methods data will inform feasibility, intervention refinement, and hypotheses regarding mental health as a mediator of HIV outcomes. Process evaluations will use the Consolidated Framework for Implementation Research (CFIR).

**Conclusions:**

This trial will assess the potential of church-based PST to address the intersecting burdens of GBV, HIV, and mental health among AGYW in Zambia. Findings will offer insights into leveraging trusted community institutions (e.g., church and religious settings) for mental health and HIV care delivery in low-resource settings.

**Trial registration:**

ClinicalTrials.gov NCT07132905. Prospectively registered on August 13, 2025. clinicaltrials.gov/study/NCT07132905.

**Supplementary Information:**

The online version contains supplementary material available at 10.1186/s13063-026-09625-3.

## Background

Young Zambian women face a dual burden of gender-based violence (GBV) and HIV, which is further compounded by mental health challenges. GBV refers to violence that is explicitly or implicitly directed at a person because of their gender and disproportionately affects women and girls [1]. GBV is inextricably linked to heightened HIV risk, primarily due to structural and social factors such as unequal gender power dynamics and patriarchal gender norms that restrict women’s ability to negotiate safe and consensual sex [2–4]. Adolescent girls and young women (AGYW), classified as women aged 15 to 24, face increased vulnerabilities. In Zambia, as in much of sub-Saharan Africa, studies consistently show that intimate partner violence (IPV), a form of GBV occurring within intimate relationships [5–7], is strongly associated with HIV seropositivity in this demographic. For instance, a pooled analysis across 30 sub-Saharan African countries, including Zambia, found that women who experienced past-year physical or sexual IPV were over three times more likely to acquire a recent HIV infection and were significantly less likely to achieve viral suppression [7]. Furthermore, physical violence, psychological abuse, or forced sex is associated with higher odds of viremia among young Zambians living with HIV [8]. GBV-exposed women are also at risk for mental health disorders including depression, anxiety, PTSD and substance misuse [5, 9–15]. GBV and mental health disorders also undermine HIV prevention and treatment engagement by decreasing antiretroviral therapy (ART) adherence, clinic attendance, and pre-exposure prophylaxis (PrEP) use [2, 8, 16–18].

Although the integration of mental health treatments within HIV interventions can lead to improvements in HIV and mental health outcomes, there is insufficient evidence of this impact among young women with a history of GBV, particularly in settings with limited human resources for health services like Zambia [19]. Mental health treatment in Zambia is constrained by limited numbers of mental health specialists such as psychiatrists, psychologists, nurses, or clinical social workers. According to the World Health Organization, Zambia has a critical shortage of mental health specialists, with approximately 0.06 psychiatrists and 1.43 mental health nurses per 100,000 people [20]. Instead, task-shifting approaches that utilize paraprofessional mental health support persons, such as psychosocial counselors, peer supporters and community health workers, have emerged as promising, cost-effective strategies for bridging the treatment gaps in behavioral and mental health care [21, 22]. This phenomenon is not unique to Zambia. Paraprofessional mental health support has successfully been used to address the mental health provider deficit in several contexts throughout sub-Saharan Africa [21, 23, 24].

Local Zambians affirm the need to leverage the sociocultural influence of the church to offer counseling and other rehabilitative services to GBV survivors [25]. Church leaders are widely trusted to provide counseling and support for GBV survivors [25]. They are often the first lines of contact in GBV-related situations in families [26]. A range of GBV-related support practices were adopted by Pentecostal churches in Lusaka to address GBV, including sponsoring couples’ meetings and home visitations, as well as providing moral, spiritual, and financial support [25]. However, both clergy and lay members emphasized the untapped potential of churches to play a more active role in offering counseling services, particularly if church leaders receive appropriate training [25, 26]. Church leaders particularly expressed interest in being trained in counseling to support women who have experienced violence [25]. Given the moral authority of religious leaders and the influence of church teachings on social norms, including norms related to GBV and HIV risk behaviors [27], the church represents a promising platform for integrated GBV and HIV interventions. Despite this potential, there remains a striking lack of rigorous evaluations assessing the effectiveness of church-led GBV-HIV programs, and evaluations of implemented GBV-HIV interventions by the church remain scarce [28]. This presents a missed opportunity to strengthen and scale up church-based GBV support for young women, who are often overlooked in existing church-based responses that focus primarily on married couples [25].

This study is a hybrid type 1 (HT1) [29] randomized controlled trial designed to evaluate the effectiveness of a church-based problem-solving therapy intervention, called *Mpata Yathu* (meaning “Our Space” in Chinyanja), for adolescent girls and young women in Zambia, while concurrently assessing implementation outcomes such as fidelity, acceptability, and feasibility. *Mpata Yathu* is an adaptation of the Friendship Bench, an evidence-based, lay-delivered problem-solving therapy (PST) intervention originally developed in Zimbabwe [30]. In this adapted version, *Mpata Yathu* is designed specifically for AGYW in Lusaka, Zambia who have experienced GBV and are either living with or at risk of acquiring HIV. The intervention will be delivered in church settings, leveraging the church’s trusted position and widespread community influence to increase accessibility, acceptability, and relevance.

This protocol outlines the objectives of the *Mpata Yathu* HT1 RCT which aim to:

- *Aim 1:* Assess the effect of the intervention on common mental health disorder (CMD) symptoms, the primary outcome for which the study is statistically powered.
- *Aim 2:* Evaluate the implementation outcomes of the Mpata Yathu intervention, specifically its feasibility, acceptability, and fidelity, as delivered by lay counselors to AGYW with a history of GBV experiences.
- *Aim 3:* Explore potential effects of the *Mpata Yathu* intervention on:
  o Additional mental health outcomes (depression, anxiety, PTSD);
  o HIV prevention and treatment engagement outcomes (based on participant HIV status);
  o GBV-related attitudes and experiences, and psychosocial variables (e.g., adverse childhood experiences and coping self-efficacy).
  o Whether changes in mental health symptoms mediate or moderate the intervention’s effects on HIV-related outcomes.

### Description of the intervention

*Mpata Yathu* is a church-based, lay counselor-delivered psychological intervention that we will evaluate in Lusaka, Zambia. The intervention is an adaptation of the Friendship Bench model. Friendship Bench, a globally recognized mental health model and originating from Zimbabwe, is a culturally responsive intervention comprising weekly PST sessions that are delivered on a bench by lay counselors who are older adult women, known as grandmothers [30]. Friendship Bench significantly reduced mean depression scores, as measured by the Patient Health Questionnaire-9 (PHQ-9) [31], by 6.36 points after a 6-month follow-up period among Zimbabwean adults (86% women, 42% HIV seropositive) [24]. The 6.36-point reduction in PHQ-9 scores is clinically meaningful because it exceeds the commonly accepted threshold for significant symptom improvement [32]. However, a cluster-randomized trial in rural Zimbabwe found that the Friendship Bench intervention significantly reduced symptoms of common mental disorders but had no significant impact on ART adherence or viral suppression among people living with HIV [33]. This suggests that while Friendship Bench is effective for mental health, additional integrated HIV care strategies (e.g., HIV linkages and follow-up) may need to be adapted to address HIV prevention and treatment outcomes among GBV survivors.

*Mpata Yathu*has been specifically tailored for AGYW aged 15 to 24 who have experienced GBV and are living with or at risk for HIV. Using principles of Community-Partnered Participatory Research (CPPR), we relied on community partner feedback to guide the adaptation process [34, 35]. In CPPR, community partners are individuals and organizations that represent and serve the community, bringing local knowledge, lived experience, and leadership to shape and guide all phases of the research [34]. The process of developing the adaptation is described elsewhere. This adaptation maintains the core principles of the original Friendship Bench intervention—namely, structured PST that supports participants in identifying and addressing personal problems—but introduces several critical modifications. These include training lay counselors on how to navigate GBV-related conversations and referrals among their clients, integrating trauma-informed and survivor-centered approaches into counseling delivery and practice (i.e., allowing clients to choose their counselor), and situating the delivery within church spaces to leverage the social authority and reach of religious institutions in Zambia. Church-based delivery is intended to reduce barriers to access by situating the intervention within trusted and familiar community environments, while also capitalizing on the role of churches as influential actors in shaping norms related to gender, mental health, and HIV.

Unlike the original model which deploys “grandmothers” to deliver the intervention, *Mpata Yathu* will be implemented by lay counselors with diverse backgrounds and age ranges (e.g., young adults, middle-aged, pastors, schoolteachers, students, peer navigators, etc.) and the clients will choose their counselor in the order of their preference. Allowing choice by the clients was intended as part of our trauma-informed, survivor-centered approach. Trained lay counselors will deliver the intervention within church settings following an intensive training program that includes PST techniques, ethical protocols, and guidance on supporting GBV survivors. Each participant will be offered six weekly sessions lasting approximately 45–60 min. In addition to PST delivery, the intervention includes structured referral pathways to mental health/psychiatric support, HIV services, and GBV response mechanisms, ensuring participants receive appropriate and timely care beyond the sessions themselves.

In a feasibility trial evaluating PST for pregnant women experiencing depressive symptoms and IPV in rural Ethiopia, participants were randomized into one of three arms: PST adapted for IPV (PST-IPV), standard PST, or enhanced usual care [36]. While the study found high acceptability and feasibility across arms, the adapted PST-IPV arm did not lead to more IPV-related content being discussed in sessions. These findings informed our decision to initially implement the standard PST model without IPV-specific modifications. However, we will monitor implementation closely and use process evaluation findings to determine whether future adaptations are necessary to better support GBV-related coping and problem-solving.

Although problem-solving therapy has demonstrated promise in improving mental health outcomes, particularly depression, among diverse populations, there remains limited evidence on its specific effectiveness for women who have experienced GBV. In a systematic review and meta-analysis, psychological interventions, including PST, were effective at reducing anxiety among women experiencing IPV in low- and middle-income countries (LMICs), even when the interventions were not specifically tailored to address IPV [37]. Another systematic review and meta-analysis reported only modest mental health benefits from psychosocial interventions with no impact on IPV recurrence [38]. Both reviews underscore the need for more rigorous, targeted research on the effectiveness and adaptation of PST for GBV survivors.

### Theoretical/conceptual model

**Fig. 1:**
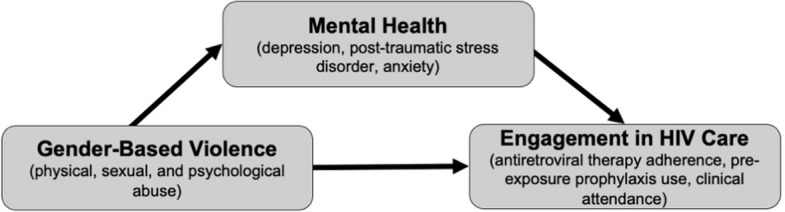
Mediating effect of mental health on gender-based violence and HIV

Conroy et al. [18] conducted a longitudinal study of over 1700 women living with HIV in the USA, and found that GBV is significantly associated with a higher odds of suboptimal medication adherence and missed clinical appointments [18]. The authors also found that mental health mediates the relationship between GBV and HIV treatment engagement. Depression, general anxiety disorder, and PTSD symptoms were found to account for 29.7%, 15.0%, and 16.5% of the total association of GBV with suboptimal adherence, respectively [18]. Adapted from Conroy et al.’s study, [18] Fig. 1 outlines mental health as a mediator of the relationship between GBV and HIV treatment engagement. Young women in Zambia exposed to sexual violence perpetrated by non-partners (i.e., men who are not boyfriends, husbands, or regular partners) have increased odds of depression and anxiety [5]. Improving mental health outcomes may therefore improve HIV treatment and prevention outcomes among these women.

### Hypothesis

We hypothesize that participation in the *Mpata Yathu* intervention may lead to improved HIV-related outcomes (e.g., increased care engagement, adherence, or testing) by reducing depressive symptoms, which are known to interfere with health-seeking behaviors and treatment adherence [39, 40]. Depression has been associated with avoidance, low self-efficacy, and disengagement from care, all of which can negatively impact HIV outcomes [41, 42]. By alleviating these psychological barriers through PST and psychosocial support, participants may improve HIV treatment self-efficacy [39, 43] and therefore become more motivated and emotionally able to engage with HIV prevention or treatment services in the absence of direct HIV-related content. Based on results from a randomized controlled feasibility trial, a PST intervention adapted for pregnant women experiencing depressive symptoms and IPV in rural Ethiopia was feasible, acceptable, and safe to deliver within antenatal care settings [36]. We anticipate that our study will demonstrate similar feasibility, acceptability, and safety when implemented in church-based settings.

Additionally, changing responses to harmful norms may serve as one solution for improving the mental health of young women who have experienced GBV [44]. For example, sexual assault survivors in the Democratic Republic of Congo were perceived as “soiled” or shameful, and these views are associated with worsened mental health outcomes among survivors [45]. Among young women living with HIV and with a history of sexual abuse, HIV-related stigma may also compound the harmful effects of sexual violence on mental health [46]. For our study in Zambia, we anticipate that the problem-solving approach of Friendship Bench will influence how young women think about and respond to normative cultural views of emotions, sexual trauma, and psychopathology [47].

We also anticipate that the PST approach will foster resilience and enhance trauma coping skills surrounding harmful social norms and environmental stressors (e.g., poverty) linked to violence against women [48, 49]. Effective problem-solving skills have been associated with helping women cope with environmental stressors related to abuse [47]. Although PST is effective for treating depression among women who have experienced IPV [47, 50], this study will assess whether PST also improves HIV treatment and prevention outcomes among GBV-exposed women.

## Methods

### Study design

**Fig. 2:**
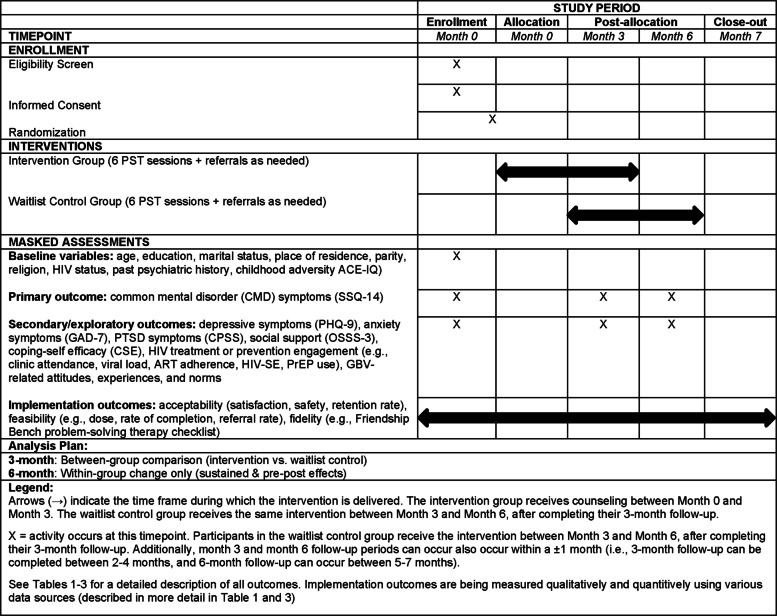
SPIRIT figure

We will conduct a two-arm HT1 randomized controlled trial (RCT) to evaluate the effectiveness and implementation of the adapted Friendship Bench intervention, *Mpata Yathu*, on HIV prevention and treatment, and depression outcomes among young women living with or at risk for HIV, with a lifetime history of GBV and depression symptoms in Zambia. This study will have two arms: an intervention group and a waitlist control group. This trial follows a superiority framework, testing whether the intervention improves outcomes compared with the waitlist control group. The SPIRIT figure [51] (Fig. 2; see Additional file 3 for Checklist) illustrates the timeline of enrollment, intervention, assessments, and close-out procedures for the RCT of the *Mpata Yathu* counseling intervention for AGYW exposed to GBV in Zambia. All items from the WHO Trial Registration Data Set are provided in Additional file 1. The intervention is designed to be delivered in six sessions, with one session per week. However, participants will be given up to 3 months to complete all sessions to accommodate common barriers such as caregiving responsibilities, stigma, mobility, and illness [52–55]. Counseling sessions will be scheduled to accommodate participant availability, and lay counselors will track session completion. After screening and enrollment, eligible participants will be randomized in a 1:1 ratio to either group.

- *Intervention group:* Participants will receive up to six individual counseling sessions between baseline and 3-month follow-up (0–3 months).
- *Waitlist control group:* Participants will receive usual care from 0 to 3 months, followed by the intervention (up to six counseling sessions) between 3 and 6 months.

Due to the nature of the intervention, participants and lay counselors will not be blinded to study arm assignment. However, quantitative outcome assessors conducting follow-up assessments will be blinded to participants’ group assignment to reduce potential bias in data collection. Unblinding will only occur in cases of adverse events or safety concerns where knowledge of intervention status is necessary for appropriate clinical management or referral.

### Study setting

Our study will be conducted in two churches in Lusaka, one Protestant church in Matero and one Anglican church in Chawama. Our community partners, who are native Zambians, recommended implementation of Friendship Bench within churches in the Matero and Chawama catchment areas of Lusaka since women are disproportionately affected by both poverty and GBV in these areas, aligning with literature linking economic vulnerability to sexual coercion [48]. The selected churches were recommended by native Zambians as appropriate sites for implementation since they serve a wide population and offer the classroom space and organizational structure needed to host counseling sessions.

### Screening and enrollment

We anticipate screening 300 young women over 4 months to enroll 90 eligible participants. We are planning for a conservative dropout rate of 30%. This estimate draws from comparable implementation research in sub-Saharan Africa, including a trial in rural Ethiopia, which used a similar assumption to test feasibility of participant retention and study procedures [56]. This estimate allows us to account for structural barriers (e.g., mobility, stigma, early pregnancy, GBV recurrence, financial hardship, caregiving responsibilities) that commonly affect longitudinal follow-up among AGYW who have experienced GBV [53, 57]. Collecting actual retention data during the RCT will allow us to empirically estimate the true dropout rate and its variability, which will inform the design and sample size calculations of a future fully powered randomized controlled trial and for refining strategies to improve participant retention.

Participants will be considered lost to follow-up for a specific assessment (at 3 or 6 months after the baseline assessment) if they cannot be reached despite at least three documented recontact attempts using various methods (e.g., phone calls, church-based outreach, or home visits, based on participant consent and preference) during the assessment window, defined as ± 1 month from the target follow-up date. Recontact efforts will begin approximately 2 weeks prior to each window and continue throughout. No outreach will occur after the close of each window. Participants who miss the 3-month follow-up assessment may still be contacted and retained for the 6-month follow-up. Participants who voluntarily withdraw from the study will not be considered lost to follow-up.

### Randomization

We will use permuted block randomization, stratified by HIV status, to ensure balance across study arms. The randomization sequence will be computer-generated using REDCap’s randomization module. REDCap will conceal allocation until after baseline data entry is complete, ensuring that research staff cannot foresee or manipulate assignments. The study biostatistician will generate the allocation sequence and upload it into REDCap. The research assistants will enroll participants, and REDCap will automatically assign the participant to their group after enrollment and baseline data completion. Participants will be randomized 1:1 to the intervention group (offering intervention sessions from 0 to 3 months) or the waitlist control group (offering intervention sessions from 3 to 6 months), with up to six sessions delivered over 3 months among both arms.

### Inclusion criteria

To be eligible for enrollment in the RCT, participants must meet the following criteria:

1. Aged 15 to 24 years;
2. Speak Nyanja, Bemba, and/or English fluently;
3. Reside in the Matero or Chawama constituency area during the time of recruitment;
4. Meet the following criteria based on selected items from the WHO Multi-Country Study on Women’s Health and Domestic Violence Against Women instrument:
  ◦ GBV Exposure Criterion (Sections 7, 8, and 10): Participant reports any emotional, physical, or sexual violence, including:
    ▪ Emotional or controlling behaviors by a current or most recent intimate partner
    ▪ Physical violence by a current or past intimate partner since age 15
    ▪ Sexual violence by a partner or non-partner since age 15
    ▪ Unwanted or coerced sexual touching or sexual acts before age 15
    ▪ Injury resulting from partner violence at any point in life
    AND/OR
  ◦ GBV Risk/Vulnerability Criterion (Sections 6 and 11): Participant demonstrates elevated vulnerability to GBV, indicated by:
    ▪ Endorsement of violence-supportive or controlling gender norms (e.g., obligation to obey husband, sexual obligation, justification of partner authority)
    ▪ Restricted financial autonomy, including partner control of earnings, financial decision-making, or ability to access money/resources
    ▪ Low confidence in the ability to secure funds independently in an emergency
5. Exhibit moderate depressive symptoms indicated by a score of 10–14 on the 9-item Patient Health Questionnaire (PHQ-9), or common mental disorder (CMD) symptoms (e.g., depression, anxiety) as indicated by a score of 9 or higher on the 14-item Shona Symptom Questionnaire (SSQ-14); and
6. Be living with HIV or demonstrate HIV risk behaviors, as defined by validated items from the World AIDS Foundation survey [58], including unprotected sex, multiple sexual partners, coerced sex, or transactional sex.

### Exclusion criteria

Women will be excluded if they:

1. Require emergency treatment for any crisis (mental, physical, emotional) at the time of screening
2. Report severe symptoms of depression (score > 14 on PHQ-9), and/or severe anxiety symptoms using the Generalized Anxiety Disorder 7-item scale or GAD-7 (score > 14 on GAD-7)
3. Have intellectual or cognitive disabilities that limit their ability to complete the screening tools, interact with a lay counselor and/or provide informed consent; and/or
4. Are considered in immediate danger (e.g., reoccurring physical violence) during time of the study.
5. Are currently receiving formal mental health counseling or psychotherapy (to avoid duplication of care and potential confounding effects).

### Study procedures for RCT participants

Recruitment, screening, and consenting will take place at both intervention and non-intervention sites across Lusaka, including churches, government health facilities, schools, and community-based organizations; the intervention will be delivered at two Catholic churches—one in Matero and the other in Chawama. These locations were identified in consultation with native Zambians who are familiar with these church settings to ensure accessibility and safety for potential participants. Private rooms within the selected locations will be used to ensure confidentiality during screening and consenting. In some cases where private rooms are unavailable, screening and consenting will take place outdoors in quiet, secluded areas to maintain privacy.

Participants will be informed whether they are eligible for the study immediately after completing the screening. Eligible individuals will be considered enrolled in the study immediately after consenting. If unavailable to screen, consent, and/or enroll during the time of recruitment, potential participants will be asked to schedule a time to complete the screening, enrolling, and/or consenting in person. The research team will conduct the screening and consenting. We will utilize the following recruitment methods:

- Passive recruitment: Study personnel will share a recruitment flyer with schools, churches, community-based organizations and clinical settings that serve AGYW, including youth-friendly spaces at government hospitals.
- Direct recruitment: Our research team will approach AGYW at selected recruitment sites (e.g., churches, schools, designated youth-friendly spaces at government hospitals during regular operating hours). Potential participants will be asked if they would like to be screened for eligibility. Interested individuals will be invited to a private or secluded space for screening by the research team, who will follow an eligibility screening script.
- Recruitment letters/flyers and referrals: Recruitment letters and flyers (via email, text, or hard copy) will be shared with church leaders, school staff, and members of community-based organizations that serve AGYW. They will be encouraged to share the recruitment flyer information within their networks or directly to any AGYW who they believe may be eligible, and/or refer women by directly contacting the study team.

Recruitment will occur over approximately 4 months (November 2025 to March 2026). Community outreach will occur through Neighborhood Health Committees (NHCs), defined as Ministry of Health (MoH)–recognized community health governance structures that link communities to local health facilities. Additional recruitment strategies will include direct approach at recruitment sites and referral partnerships with community organizations. To enhance recruitment and retention, flexible scheduling, reminder phone calls, church-based follow-ups, and participant-preferred contact methods will be used. Participants may complete sessions within a 3-month window to accommodate mobility, caregiving, or school responsibilities, and stigma-related barriers.

Consenting will be conducted by the local study team, including pastors, students, teachers, and youth peer navigators, who have completed training in research ethics and informed consent procedures. While some lay counselors delivering the intervention may also assist with consenting, they will not administer baseline or follow-up questionnaires to avoid potential bias in participant responses. All study personnel, including the lay counselors, will be certified in human subjects protection prior to study launch and follow safety protocols for women who may still be experiencing GBV and/or have specific mental health needs outside of the scope of our study (e.g., severe depression, suicidal ideation or attempt, psychosis).

### Study outcomes

All outcomes will be assessed at both 3-month and 6-month follow-up timepoints. The 3-month follow-up will serve as the primary endpoint for between-group comparisons (intervention vs. waitlist control). At the 6-month follow-up, only within-group changes will be assessed, as both groups will have received the intervention by this time. The waitlist control group will begin intervention delivery after completing the 3-month assessment, with no contamination expected due to the ± 1 month window applied to the time in which follow-up assessments must be completed. We outline all quantitative study outcomes with their corresponding measures and timepoints in Table 1. Table 2 outlines the HIV prevention and engagement outcomes, which are reported separately since outcome measures differ based on seropositivity status. Table 3 describes the qualitative measures, which are all implementation process measures. All instruments will be translated from English to Nyanja and Bemba using a forward–backward translation process to ensure linguistic and cultural equivalence.

**Table 1:** Quantitative outcomes, measures, and data collection timeline (excluding HIV outcomes)

Outcome	Measure or data source	Data collection time points		
		Baseline/pre-intervention	3-month follow-up	6-month follow-up
**Mental health symptoms**				
***Common mental disorder symptoms***	Shona Symptom Questionnaire-14 (SSQ) [59]	X	X	X
***Depressive symptoms***	Patient Health Questionnaire-9 (PHQ-9) [31]	X	X	X
***Anxiety***	Generalized Anxiety Disorder (GAD-7) [60]	X	X	X
***Post-traumatic stress disorder symptoms***	Child PTSD Symptom Scale (CPSS) [61]	X	X	X
***GBV experiences and perceptions***				
***GBV experiences and attitudes***	WHO Multi-country Study on Women’s Health and Domestic Violence Against Women Instrument [1]	X	X	X
***GBV social norms and beliefs***	Perrin et al.’s Social Norms and Beliefs about Gender-Based Violence Scale [62]	X	X	X
***HIV engagement***				
***Treatment engagement (if HIV positive)***	See Table 2 for specific measures	X	X	X
***Prevention engagement (if HIV negative/status unknown)***	See Table 2 for specific measures	X	X	X
**Feasibility**				
***Recruitment time***	Time required to recruit clients and counselors (days)	X		
***Recruitment rate***	Number of eligible participants required to reach desired sample size for clients	X		
***Strictness of eligibility criteria***	Reasons for non-participation among eligible participants	X		
***Sample characteristics of clients***	Demographics of clients (e.g., age, urban/rural residence, HIV status)	X		
***Dose***	Number of sessions attended by each participant (mean number of sessions attended per arm)		X	X
***Rate of completion***	% of participants who completed all 6 sessions			X
***Time of completion***	Time required for participants to complete all aspects of the intervention (days)			X
***Referral rate***	The number of individuals referred to indicated mental health, HIV, and/or GBV services out of all individuals screened	X	X	X
**Acceptability**				
***Retention rate***	Number of clients and counselors who drop out of the intervention	X	X	X
***Safety***	Number of adverse events reported to the IRB	X	X	X
***Satisfaction***	Acceptability of Intervention Measure [63]		X	X
**Fidelity** ***Adherence to PST techniques***	Friendship Bench Fidelity Checklist assessed via audio recordings (if consented) ^‡^			
**Psychosocial**				
***Adverse childhood experiences***	Adverse Childhood Experiences International Questionnaire (ACE-IQ) [64]	X		
***Resilience***	Coping Self-Efficacy (CSE) [65]	X	X	X
***Social Support***	Oslo Social Support Scale (OSSS-3) [66]	X	X	X

‡To be evaluated throughout the intervention period

**Table 2:** Summary of measures for HIV-related outcomes

Outcome	Subgroup	Measurement type	Specific measures
**HIV treatment engagement**	Participants living with HIV	Self-report (behavioral)	Clinic attendance in past 6 months (noting visit frequency may vary by treatment regimen)
		Clinical (optional, if shared)	Most recent viral load; ART regimen; pharmacy refills/missed doses (if noted); clinic attendance documented in health passport or personal medical record
		Psychosocial (self-efficacy)	HIV Self-Efficacy Questionnaire (HIV-SE) [67] to assess confidence in engaging in care
**HIV treatment engagement**	Participants newly diagnosed during study	Self-report	Date and location of most recent HIV test; linkage to clinic/facility for confirmatory testing; referral source (e.g., study team); ART initiation and timing
**HIV prevention engagement**	Participants not living with HIV	Self-report (baseline eligibility)	Sexual Behavior Practices subscale from WAF Survey [58] (e.g., # of partners, condom use, coercive sex, PrEP use)
		Self-report (follow-up)	Repeat WAF items to assess change: condom use consistency, PrEP uptake, reduction in coercive sex or high-risk behaviors
		Psychosocial (readiness)	HIV Prevention Readiness Measure (HPRM) [8]: subscales on self-efficacy, disclosure, and social support; measures readiness and sustained engagement in prevention

**Table 3:** Qualitative implementation process measures

Domain	Definition	Data source
Burden	Risks and benefits associated with participation among counselors and study participants	Qualitative interviews with counselors and participants (post-intervention)
Participant responsiveness	Participant engagement and receptiveness to the intervention	Post-session counselor checklist and logs (during intervention), debrief notes (during intervention)
Content coverage	Emergent discussion of key psychosocial themes (e.g., past GBV, HIV-related stressors)	Session summaries and audio reviews (if available) (during intervention, debrief notes (during intervention)
Barriers and facilitators	Factors affecting delivery and engagement (e.g., stigma, trust, mobility)	Qualitative interviews with counselors and participants (post-intervention), debrief notes (during intervention)
Satisfaction	The extent to which counselors and study participants will participate in the intervention again	Qualitative interviews with counselors and participants (post-intervention), Acceptability of Intervention Measure [63]

#### Mental health outcomes

The primary outcome is symptoms of common mental disorders (CMDs), assessed using the Shona Symptom Questionnaire (SSQ-14) [59]. The SSQ-14 consists of 14 yes/no items assessing psychological distress, with a score ≥ 9 indicating probable CMD. This outcome will be measured at 3 months and analyzed as a continuous variable, consistent with the original Friendship Bench study, which used SSQ-14 scores as a continuous measure to assess symptom reduction overtime [24]. The SSQ-14 was selected as the primary outcome measure because it is a culturally adapted and validated screening tool developed specifically for Zimbabwean and similar Southern African primary care populations. It captures symptoms of CMDs across emotional, somatic, and functional domains, and demonstrated strong psychometric properties in the original Friendship Bench trial [24].

The secondary outcome is depressive symptoms, measured using the Patient Health Questionnaire-9 (PHQ-9). PHQ-9 scores will also be analyzed using both binary and continuous analysis, consistent with the original Friendship Bench trial [24]. Although eligibility for this study includes a PHQ-9 score between 10 and 14, PHQ-9 will also be analyzed as a binary variable using a cutoff of ≥ 11 to estimate the prevalence of probable depression, as done in the original trial [24]. The PHQ-9 was selected as the secondary outcome due to its global recognition and its use in clinical settings in Zambia. Using the PHQ-9 enables broader comparability with global mental health studies and potential clinical integration. Exploratory mental health outcomes include anxiety and PTSD symptoms, assessed with the Generalized Anxiety Disorder (GAD-7) scale and Child PTSD Symptom Scale (CPSS) [61], respectively. CPPS was validated among children and adolescents in Zambia with good internal reliability (*α*> 0.80) [68].

#### HIV outcomes

All HIV-related outcomes (treatment and prevention) are exploratory but will be measured at baseline and at 3- and 6-month follow-up. All HIV-related outcomes (for prevention and treatment) are described in Table 2. We will assess HIV prevention engagement and risk behaviors among participants in our study who are not living with HIV, using a self-reported survey on HIV risk behaviors using measures from the World AIDS Foundation (WAF) Survey [58]. These questions will serve a dual purpose:

- *Eligibility Screening:* At baseline, a brief risk assessment will be administered during the eligibility screening phase to identify young women who are at heightened risk for HIV acquisition. Items will be drawn from the Sexual Behavior Practices subscale of the WAF, a validated tool for assessing HIV-related risk behaviors in sub-Saharan African contexts [58]. Specifically, we will include questions that assess the number of lifetime and recent sexual partners, frequency of unprotected sex or inconsistent condom use, experiences of coerced or forced sex, and use of pre-exposure prophylaxis (PrEP) or other HIV prevention strategies. These items align with high-sensitivity indicators of HIV risk and have demonstrated discriminant validity among youth populations in Zambia [58].
- *Follow-Up Assessment:* The same set of risk behavior items will be administered again at follow-up visits (e.g., 3- and 6-month post-intervention) to assess changes in HIV risk behaviors and engagement in HIV prevention strategies (e.g., consistent condom use, PrEP uptake, and reduced exposure to coercive sex).

We will also administer the HIV Prevention Readiness Measure (HPRM) [69] at both baseline and follow-up. The HPRM is a validated instrument originally developed for the HPTN 082 study to assess readiness for and sustained use of HIV prevention strategies such as PrEP among AGYW in sub-Saharan Africa [69]. It includes subscales on self-efficacy, disclosure, and social support, which are strong indicators of behavioral and psychosocial engagement in HIV prevention.

HIV treatment engagement will be assessed among participants living with HIV through both self-reported and clinical measures. We will distinguish between women who are already diagnosed with HIV and those who may be newly diagnosed during the study period.

1. For women already diagnosed with HIV, we will assess:
  o Self-reported clinic attendance over the past 6 months, recognizing that visit frequency may vary by treatment regimen and differentiated service delivery model (e.g., 1-, 3-, or 6-month refill cycles).
  o Where available, participants will be asked to voluntarily share their health passport or personal medical record to assess additional treatment engagement indicators such as:
    ▪ Most recent HIV viral load
    ▪ ART regimen
    ▪ Pharmacy refills or missed doses (if noted)
    ▪ Clinic attendance
2. For women newly diagnosed during the study, we will use the following engagement indicators to assess initial linkage to care:
  o Date and location of most recent HIV test
  o Whether and where they linked to a clinic or facility for confirmatory testing and care
  o Whether they were referred to care by a member of the study team
  o Whether ART was initiated, and how soon after diagnosis it began

Lastly, HIV treatment self-efficacy among seropositive participants will be assessed using the HIV Self-Efficacy Questionnaire (HIV-SE) [67], which reflects participants’ confidence in managing their HIV care and may be correlated with treatment engagement.

#### GBV experiences, attitudes, and perceptions

GBV-related experiences, attitudes and perceptions will be measured as exploratory outcomes. Experiences of GBV will be assessed using the WHO Multi-country Study on Women’s Health and Domestic Violence Against Women Instrument [1] (Sections 7–10: physical, sexual, and emotional violence by partners and non-partners), while attitudes and risk perceptions will be evaluated using Section 6 of the same instrument. GBV perceptions will also be measured using Perrin et al.’s Social Norms and Beliefs about Gender-Based Violence Scale [62]. These measures were selected for their strong psychometric properties and relevance to sub-Saharan African contexts. Including both experience- and perception-based measures will allow us to assess participants’ attitudes and beliefs, which may serve as important indicators of GBV risk.

#### Implementation outcomes

We will quantitatively assess feasibility outcomes, acceptability, and fidelity. All feasibility metrics are summarized in Table 3. Acceptability will be measured by evaluating retention rate (i.e., number of participants and lay counselors who drop out of the intervention) and safety (i.e., number of adverse events reported to the Institutional Review Board). To assess the acceptability of the intervention from the perspective of participants, we will use the Acceptability of Intervention Measure [63]. AIM is a 4-item scale that captures participants’ overall perceptions of the intervention’s acceptability, using a 5-point Likert response format. These items assess whether participants found the intervention appealing, satisfactory, and appropriate. Acceptability will also be measured through semi-structured interviews with participants and lay counselors as described below. This mixed-method approach (close- and open-ended items) will provide both quantitative and qualitative data on acceptability and identify areas for improvement in future implementation. All qualitative metrics are captured in Table 3.

Lay counselor adherence to the protocol will be monitored using a fidelity checklist and weekly supervision sessions. Fidelity will capture lay counselors' adherence to core problem-solving therapy techniques. Fidelity will be assessed through audio recordings of counseling sessions, contingent upon participant consent, as individuals may choose to opt out of being recorded. During the initial phase of implementation (within the first 2 weeks), all the sessions will be reviewed in a timely manner to provide formative feedback and support counselor development. A random sample of approximately 20% of recorded sessions will be evaluated thereafter.

Lastly, we will qualitatively assess barriers and facilitators of Friendship Bench implementation via semi-structured interviews on intervention experiences among study participants and lay counselors using relevant domains and constructs from the Consolidated Framework for Implementation Research (CFIR) [70], and the modified CFIR for LMICs [71]. We are hoping that capturing barriers and facilitators would also allow us to qualitatively assess satisfaction and burden (i.e., risks and benefits associated with participation among counselors and clients). We will qualitatively assess the feasibility and acceptability of the intervention through semi-structured interviews with both participants and lay counselors. All lay counselors who delivered the intervention (up to 12 total) will be invited to voluntarily participate in an interview. Additionally, we will conduct up to 15 interviews with participants from each study arm (intervention and delayed control group) to gather diverse perspectives on the intervention experience. To accommodate participants who are not fluent or comfortable communicating in English, interviews will be conducted in local languages (e.g., Nyanja or Bemba) by trained bilingual interviewers. All audio recordings will be transcribed and then translated into English (if applicable) for analysis. To ensure accuracy and cultural validity, a subset of transcripts will undergo back-translation and review by the research team.

We will also qualitatively evaluate content coverage of sessions which will include documentation of whether counselors explore topics such as interpersonal challenges (e.g., family or peer relationships), past (not current) experiences of violence, suicidal ideation or distress, and HIV treatment or prevention challenges. These areas are consistent with known stressors among the target population and align with the intervention’s goals of improving coping and mental health outcomes. Content coverage will be assessed as a process measure (i.e., what topics came up in sessions), and as an implementation determinant (i.e., why some topics were avoided or emphasized), especially based on lay counselor interviews or debriefs. Content coverage will be covered in the following ways:

- *Lay counselor post-session summary forms:* After each session, lay counselors will complete an open-ended note summarizing the main issues discussed. These can be thematically coded by the research team.
- *Qualitative debriefs or supervision logs:* Counselors may discuss recurring content during weekly group supervision meetings which can be documented and analyzed for patterns.
- *Lay counselor semi-structured interviews:* As mentioned above, up to 12 lay counselors will participate in semi-structured interviews which may also capture content coverage discussed.

Participant responsiveness will be qualitatively assessed through multiple sources. First, we will conduct semi-structured interviews with both lay counselors and participants to explore how participants experienced the Friendship Bench sessions, which may give us insight on their level of engagement, perceived relevance of the content, and emotional or behavioral reactions. Second, we will analyze post-session summary notes completed by lay counselors after each session, which include reflections on how engaged the participant appeared, whether they participated in problem-solving activities, and how they responded to the session content. Additionally, study team debrief notes—documenting informal observations and key discussion points during supervision meetings—will be reviewed to triangulate findings and assess patterns of responsiveness across participants. This multi-source, qualitative approach will provide a rich understanding of participant responsiveness and help inform adaptations to enhance engagement in future implementation.

#### Psychosocial outcomes

All psychosocial measures are exploratory. We will include the Adverse Childhood Experiences International Questionnaire (ACE-IQ) [64] and the Coping Self-Efficacy (CSE) [65] scale as exploratory psychosocial measures. The ACE-IQ assesses exposure to early life adversity, providing context for participants’ mental health needs and potential barriers to care. The CSE scale measures perceived ability to cope with stress through problem-solving, emotional regulation, and social support. Lastly, we will be measuring social support using the 3-item Oslo social support scale (OSSS-3) [66], which measure the number of close confidants, perceived concern from others, and ease of obtaining practical help. Together, these tools will help us explore how early adversity and coping capacity may influence engagement with the intervention and inform future trauma- and resilience-informed adaptations.

### Power analysis

This RCT is powered only to detect intervention effects on symptoms of CMDs, as measured by the SSQ-14, which is the primary outcome. All other quantitative outcomes—including other mental health measures, HIV engagement indicators, and psychosocial variables—will be treated as exploratory and used to inform effect size estimates and future trial design. With the proposed sample size of 126 participants (63 per arm) and using a two-sided alpha level of 0.05, the study will have 80% power to detect a medium effect size of Cohen’s *d* = 0.5 in SSQ-14 scores, which will be analyzed as a continuous outcome. This is a conservative estimate, selected to reflect a clinically meaningful but smaller effect than that observed in the original Friendship Bench trial, which reported a large effect (*d* ≈ 0.75–0.8) over a 6-month period. Accounting for 30% attrition, the sample size estimate was adjusted to account for an original sample size of 180 participants (90 per group). Since our study primarily measures outcomes at 3 months, a smaller effect size is assumed to avoid overestimating power. Between-group comparisons will be conducted at the 3-month follow-up, prior to the waitlist control group receiving the intervention. At 6 months, only within-group analyses will be conducted to assess sustained changes in the intervention group and pre-post change in the waitlist control group. No between-group comparisons will be performed at 6 months.

### Quantitative data analysis

We will use descriptive statistics to summarize participant characteristics at baseline, feasibility and acceptability metrics, and other implementation outcomes. To assess baseline differences between participants who complete the intervention and those who drop out, we will conduct bivariate comparisons using independent samples t-tests or Wilcoxon rank-sum tests for continuous variables and chi-square or Fisher’s exact tests for categorical variables.

*Primary outcome analysis (powered):* To assess the effect of the adapted intervention on symptoms of CMDs (SSQ-14), we will use mixed-effects linear regression models. This is the only outcome for which the study is statistically powered, based on detecting a standardized mean difference of Cohen’s d = 0.5 at the 3-month follow-up. The model will include:

- A random intercept for participants to account for within-subject correlation from repeated measures;
- A fixed effect for time (baseline vs. follow-up);
- A fixed effect for study group (immediate intervention vs. delayed control);
- A group × time interaction term to estimate the differential intervention effect over time.

If the model assumptions (e.g., homoscedasticity, normally distributed residuals) are not met, we will consider using generalized linear mixed models with appropriate link functions and distributional assumptions to reduce the risk of Type I and Type II errors.

*Exploratory analyses:* All other outcomes will be analyzed using mixed-effects linear, logistic, or ordinal regression models, depending on the outcome type. These outcomes include:

- Mental health outcomes: depression (PHQ-9; binary and continuous), anxiety (GAD-7; continuous), and PTSD (CPSS; continuous) symptoms;
- HIV prevention outcomes (among HIV-negative or status-unknown participants): consistent condom use, PrEP uptake or readiness, HIV risk behaviors (via WAF), and HIV prevention self-efficacy/readiness (via HPRM);
- HIV treatment outcomes (among HIV-positive participants): ART adherence, clinic attendance, viral load documentation (if available), and HIV treatment self-efficacy (via HIV-SE);

*Composite HIV engagement scores*: To simplify analysis and reflect multiple components of HIV prevention and treatment engagement, we will construct composite engagement scores for each group (based on HIV status). Composite scores will be created by assigning a binary value (e.g., 0 = no, 1 = yes) to multiple related indicators of HIV engagement, then adding the values together to generate a single continuous score. These composite scores will be analyzed as exploratory outcomes, included as covariates in models predicting mental health outcomes, and used to test for interaction effects with intervention group to explore potential moderation.

- For HIV treatment engagement (among participants living with HIV), the following indicators will be composited into a single score:
  - ◦ Self-reported clinic attendance;
  - ◦ ART initiation and adherence;
  - ◦ HIV viral load documentation;
  - ◦ HIV treatment self-efficacy (via HIV-SE).
    ◦ Each indicator will be scored and summed to create an overall treatment engagement score.
  - ◦ Higher scores reflect stronger engagement in HIV care.
- For HIV prevention engagement (among HIV-negative or status-unknown participants), the following indicators will be composited:
  - ◦ Consistent condom use;
  - ◦ PrEP uptake or readiness;
  - ◦ Reduction in HIV risk behaviors (via WAF);
  - ◦ HIV prevention self-efficacy/readiness (via HPRM).
  - ◦ Higher scores reflect stronger engagement in HIV prevention strategies.

*Moderation and mediation analyses (exploratory)*: Post hoc exploratory analyses will assess whether changes in mental health outcomes mediate or moderate the intervention’s effects on HIV-related outcomes. Mental health change scores will be calculated separately for each follow-up period (i.e., 3 months and 6 months post-intervention) by subtracting baseline values from the respective follow-up values. This will allow us to assess both short-term and sustained mental health changes and explore their relationship with HIV-related outcomes over time.

- Mediation: We will test whether changes in CMD symptoms (SSQ-14), depression (PHQ-9), anxiety (GAD-7), or PTSD (CPSS) symptoms help explain (mediate) observed changes in HIV prevention or treatment outcomes using causal mediation models.
- Moderation: We will include interaction terms between changes in mental health symptoms and intervention group to assess whether the impact of the intervention on HIV outcomes differs depending on mental health changes. Additionally, we will explore whether baseline levels of early adversity (measured by the Adverse Childhood Experiences International Questionnaire; ACE-IQ) [64], social support (measured by the Oslo social support scale; OSSS-3) [66], and coping self-efficacy (measured by the Coping Self-Efficacy scale; CSE) [65] moderate the relationship between the intervention and mental health or HIV outcomes.

*Adjustment variables and stratified analyses:* HIV status (positive vs. negative/unknown) will be included as a covariate in pooled models to adjust for baseline differences. Stratified analyses by HIV status will be considered if each group has a sufficient sample size to support reliable subgroup modeling (e.g., ≥ 40 participants per group). Given the small overall sample size (*N* = 90, accounting for 30% attrition), such stratified analyses may be underpowered and are therefore considered exploratory and hypothesis-generating only. All models will follow an intention-to-treat (ITT) framework, and statistical significance will be assessed at a two-sided *α* = 0.05, with 95% confidence intervals reported. Non-adherence will be handled by analyzing all participants as randomized (ITT); we will also run a pre-specified per-protocol sensitivity analysis defined as completion of ≥ 4/6 sessions and no major protocol deviations. Missing data will be addressed using mixed-effects models, which provide valid estimates under missing-at-random assumptions.

### Qualitative data analysis

We will utilize relevant constructs from the original CFIR developed by Damschroder and colleagues [70] to guide a thematic analysis using deductive and inductive approaches. CFIR is a meta-theoretical framework with 39 constructs synthesized from multiple implementation theories. We will also incorporate *community characteristics*, a novel construct that falls under the Outer Setting CFIR Domain, proposed by Means and colleagues [71] to consider while conducting implementation research in LMICs. Community characteristics include sociocultural and religious features of the clients and/or their parents, health knowledge, attitudes, and beliefs influencing demand for healthcare services [71]. Our analysis will focus on the following CFIR domains and corresponding constructs:

- Intervention characteristics (relative advantage, complexity, cost)
- Outer setting (patient needs and resources, community characteristics)
- Inner setting (culture, implementation climate, readiness for implementation)
- Individual characteristics (knowledge, beliefs, self-efficacy to deliver)
- Process (planning, opinion leaders, champions)

The above CFIR domains and constructs will be prioritized for analysis based on their theoretical and contextual relevance to the intervention model and church-based implementation setting. Interview transcripts will be analyzed in two stages. In the first stage, the first transcript (i.e., the pilot transcript) will be independently coded by two researchers to establish a consistent coding strategy and address any challenges related to applying the CFIR constructs. The preliminary results from the pilot transcript coding will be reviewed and discussed before proceeding to code the remaining transcripts and to develop a codebook. In the second stage, the remaining transcripts will be coded independently by the same two researchers using ATLAS.ti software and the finalized codebook. This process will involve multiple readings of the transcripts, which will be deductively coded according to the selected CFIR constructs above. Inductive coding will be used for emerging themes that were not expected.

Coded data will then be grouped into themes and sub-themes within each CFIR domain, and representative quotes will be identified to support key findings. We will remain open to emergent codes and themes that do not fit within the CFIR framework to ensure that context-specific insights are not overlooked. These inductively identified codes will be documented separately and, where appropriate, integrated into the final thematic structure. Throughout the process, coding disagreements will be resolved through discussion or consultation with a third reviewer. To ensure rigor, inter-coder agreement will be assessed at multiple points. We will use a consensus-building approach, where the coders meet regularly to compare coding decisions and resolve discrepancies through discussion. If disagreements persist, a third reviewer will be consulted. We will also calculate Cohen’s kappa as a statistical measure of inter-coder reliability, aiming for a kappa score of ≥ 0.80, which reflects strong agreement and meets accepted standards for health research [72]. Finally, to enhance cultural validity and relevance, we will review preliminary themes with lay counselors who delivered the intervention and integrate their feedback into our final interpretation of the findings.

### Ethical considerations

#### Ethics approval

This protocol will be reviewed and approved by the following bodies: University of California, Los Angeles (UCLA) Institutional Review Board and the University of Zambia Biomedical Research Ethics Committee (UNZABREC). Ethical approval includes clearance from Zambia’s National Health Research Authority (NHRA), which allows for recruitment and data collection at government healthcare facilities. Any important protocol modifications (e.g., changes to eligibility criteria, outcomes, or analyses) will be submitted for approval to the UCLA IRB and UNZABREC.

#### Referral and safety protocols

The adapted intervention includes a built-in referral pathway to connect participants with available HIV- and GBV-related services. This ensures ethical linkage to care for individuals experiencing ongoing violence or HIV risk. Our referral pathway is integrated into the adapted intervention design. Lay counselors and study staff will be equipped to refer participants to existing HIV- and GBV-related resources such as One Stop Centres and clinic-based HIV counseling and testing services. One Stop Centres are facilities—often located within government hospitals or clinics—that offer comprehensive services for individuals affected by GBV, including medical care, psychosocial support, legal assistance, and police services. Given recent reductions in U.S.-funded HIV and GBV programs, the availability of services may vary. Therefore, referral pathways will be documented and regularly reviewed in collaboration with local partners to ensure appropriate linkage to care. This referral process aligns with our ethical responsibility to support participants beyond the scope of counseling sessions.

Similar to the pilot trial of the Friendship Bench in Zimbabwe, participants who present with suicidal ideation or do not show clinical improvement during or after the six sessions will be referred to a mental health clinician or supervisor for additional support and possible referral to specialized care. In the original pilot, fewer than 10% of individuals who screened positive for common mental disorders were referred to a mental health specialist, primarily due to suicide risk or very severe symptoms identified at screening [73]. Additionally, we will record all safety events such as suicidality, worsening symptoms, and non-suicidal self-injury (i.e., self-cutting and burning). Though not likely, if rates of suicidal ideation increase beyond baseline levels, there will be an automatic study pause for ethics review. Stopping rules include halting the study in cases of severe emotional distress among 10% of participants or more at any point during implementation of the study, unforeseen harm, ethical or legal violations, or when potential harm outweighs benefits.

Adverse events will be documented by study staff and reviewed by the PI. Serious adverse events, including suicidality, will trigger immediate referral per the safety protocol and be reported to the UCLA IRB and UNZABREC.

#### Informed consent and risk minimization

Informed consent will be obtained by trained local study staff (e.g., pastors, teachers, students, youth peer navigators) who have completed human subjects protection training. Consent discussions will take place in private settings and in the participant’s preferred language (English, Nyanja, or Bemba) using IRB-approved consent forms. Participants under the age of 18 will not be required to obtain parental or guardian consent due to potential histories of abuse within the home. Participants aged 15–17 years will provide written informed assent; parental consent has been waived by the UCLA and University of Zambia IRBs due to potential histories of abuse in the home. The consent process will emphasize the participants’ right to make their own decisions about participating. A model informed consent form is available as a supplementary file.

Participants may discontinue the intervention if they experience distress or upon personal request. Lay counselors may also discontinue sessions and initiate referrals if participants disclose imminent harm or require higher-level psychiatric care. The potential risks and discomforts associated with the research study include emotional distress and the reawakening of past trauma, particularly when discussing sensitive topics like GBV, mental health issues, and HIV. To minimize these risks, participants will be informed about the nature of the study in the informed consent process. Additionally, participants can choose to skip questions or discontinue their participation at any time if they feel uncomfortable.

#### Religious context and setting

Another important ethical consideration involves the potential for re-traumatization among women who have experienced GBV or other forms of abuse within church settings or other religious contexts. While the church-based model is intended to leverage trusted community structures, we recognize that for some participants, these spaces may evoke discomfort or mistrust. Furthermore, theological interpretations within church settings may inadvertently perpetuate silence or acceptance of violence within marriages, particularly against women [25]. To mitigate these risks, we have already engaged in conversations with local Zambians to weigh the potential harms and benefits of a church-based setting. These discussions acknowledged that while churches have been sites of harm for some, they are also widely viewed as spaces of healing and safety in Zambia, a predominantly Christian country. In addition, we are partnering with a local organization that has expertise in preventing and combatting church-related abuse in Zambia, ensuring that participant safety and trust remain central throughout implementation.

#### Incentives and reimbursements

To promote adherence, participants receive modest reimbursements for each counseling session attended and for follow-up assessments. Adherence to the intervention will be monitored by lay counselors, who will track the number of sessions completed using standardized logs.

#### Data privacy, protection, and sharing

All participant information will be de-identified by assigning subject ID numbers in place of names. Lay counselors will sign confidentiality agreements, and data will be stored securely using REDCap. Audio recordings, accessible only to the study team, will be destroyed 2 years after the study ends. We will publish study findings without using participant names. All quantitative data will be entered into REDCap. Data entry will include built-in range checks in REDCap, and research staff will conduct double-checking for accuracy.

Access to the final trial dataset will be restricted to the Principal Investigator and designated study team members. There are no contractual agreements that limit investigator access. After study completion, de-identified data may be made available upon reasonable request to the corresponding author with appropriate ethical approvals.

Study oversight is maintained by the PI (the first author) and IRBs. There is no separate steering committee or endpoint adjudication team for this trial. The PI and Co-Investigators will oversee all aspects of implementation and data management. Internal auditing will be conducted by the research coordinator, with oversight and periodic review by the PI to ensure protocol adherence and ethical conduct. No independent data monitoring committee (DMC) is used for this RCT given its small sample size and minimal risk design.

#### Risk-benefit analysis

We determined that the risks involved in study participation are reasonable in relation to the anticipated benefits and the significance of knowledge gained. The potential benefits to participants are improved mental health and enhanced HIV prevention and treatment outcomes, which are particularly important given the high prevalence of these issues in the target population. The study has the potential to provide valuable insights into the integration of a mental health intervention for young women who have experienced GBV, potentially leading to better support and care for affected individuals.

#### Dissemination

Results will be disseminated to participants, investigators, and the public through reports, peer-reviewed publications, and ClinicalTrials.gov; there are no publication restrictions.

## Discussion

Our RCT will provide several implications regarding the intersection of violence against women, mental health, and HIV/AIDS risk in Zambia, which may be applied to similar contexts. We will implement an evidence-based intervention in a non-traditional space—the church. By situating our intervention within churches, we aim to harness the trust and interpersonal bonds within these communities while using trauma-informed, survivor-centered approaches to account for potential harms. We anticipate that our findings will provide new evidence about the importance of culturally sensitive approaches that leverage existing community strengths and structures to address GBV and its intersection with HIV.

Potential barriers to implementation may include stigma related to mental health, HIV, and experiences of violence, as well as concerns about privacy and safety when disclosing sensitive information. We plan to enforce confidentiality and create safe, trauma-informed spaces for participants to minimize these risks. Additionally, dropout in our setting may be influenced by cultural norms around GBV disclosure and safety concerns. Rapid fluctuations in local HIV service delivery programs and accessibility of care through U.S. government-funded programs like PEPFAR and USAID will undoubtedly impact uptake and retainment in HIV services. Tracking dropout patterns and HIV linkages, as well as any system-level changes to service delivery, will help us assess not only individual retention strategies but also broader issues of HIV system resilience.

This study may also reveal the potential barriers of using paraprofessional models to deliver mental health interventions to GBV survivors. While engaging lay counselors can improve accessibility and cultural relevance, challenges include ensuring adequate training, supervision, emotional support for counselors, and long-term sustainability in under-resourced health systems. In an adapted version of Friendship Bench in Botswana for adolescents living with HIV involving peer delivery of the intervention, lay counselors identified concerns regarding shared trauma between themselves and their clients [74]. We also acknowledge that our current intervention may not sufficiently address the specific trauma and needs of GBV survivors, such as structural and relational factors contributing to ongoing violence. A qualitative study of a group-based participatory intervention, known as Stepping Stones, showed that the program fostered self-reflection and shifts in gender norms and relationship behaviors among rural South African youth, though broader structural challenges limited consistent behavior change related to violence, sexual health, and gender norms [75].

### Future directions

As we continue to evaluate and refine the *Mpata Yathu* intervention, we will consider developing and validating a structured tool to assess participant engagement during counseling sessions. While fidelity checklists capture adherence to core intervention components, they often overlook the relational and affective dimensions of session quality. Recent digital innovations, such as Hartmann et al.’s [76] development of a relationship counseling website to mitigate IPV in the context of HIV prevention, highlight the importance of incorporating structured engagement and relational quality measures into gender-sensitive health interventions. Therefore, we may explore digital adaptations (e.g., mobile or tablet-based) to support real-time supervision, feedback, and quality improvement. We would also like to explore creating predictive models to determine subgroups of AGYW who are likely to be most at risk for HIV treatment disengagement and/or reoccurring violence.

Our local community partners also recommended future implementation of the Friendship Bench intervention within school settings. As corroborated by the literature [77, 78], our community partners considered schools as another setting for promoting GBV prevention and supporting GBV-exposed women because 1) the school provides a structured environment conducive to delivering consistent interventions, and 2) teachers and school counselors can be trained to mediate and identify signs of GBV. A school-based intervention in South Africa demonstrated efficacy in protecting against GBV [79]. However, evidence on the provision of GBV-based counseling in schools remains limited. Additionally, there is existing policy to support school-based GBV interventions in Zambia, but with inconsistent implementation [80].

Given the limited number of GBV screening tools validated in the context of our study setting, we would like to explore validation of the WHO Multi-country Study on Women’s Health and Domestic Violence against Women questionnaire [1], which is the tool we are using for screening, baseline, and follow-up in this study. While the WHO questionnaire has been validated in several countries, including settings within sub-Saharan Africa, it has not been formally validated in Zambia. A local validation study would allow us to assess the cultural relevance, linguistic appropriateness, and psychometric properties of the tool in the Zambian context. As an exploratory analysis, we could also examine whether responses to the tool’s risk perception questions (e.g., fear of partner, perceived likelihood of future violence) are predictive of actual exposure to physical, sexual, or emotional violence. This will help assess the potential of risk perception as an early indicator of GBV and contribute to understanding the tool’s construct validity in the Zambian context.

## Conclusion

We discussed our protocol for a two-arm RCT of a PST intervention, *Mpata Yathu,* adapted from the Friendship Bench model, implemented in church settings to address syndemic outcomes (i.e., HIV and mental health disorder comorbidities) among AGYW with GBV histories. We anticipate that the results of our study will inform the development of larger, fully powered randomized trials of the church-based PST for GBV survivors in Zambia. Our study will also determine the feasibility and appropriateness of continuing such GBV-based interventions within church settings. Our study may also provide insight into the role in which mental health outcomes (e.g., depression, anxiety, PTSD) and psychosocial outcomes (e.g., childhood adversity and coping self-efficacy) may moderate or mediate the relationship between GBV experiences and HIV prevention and care engagement. We also hope that our trial will lead to further refinement and improvement of *Mpata Yathu* to address the unique needs and challenges faced by GBV survivors in culturally appropriate ways in Zambia and related contexts.

## Supplementary Information

Additional file 1. Label as WHO Trial Registration Data.

Additional file 2. Label as informed consent form template.

Additional file 3. SPIRIT checklist.

## Data Availability

Access to the final de-identified dataset will be restricted to the Principal Investigator (PI, first author) and designated study team members. External requests for data will be considered on a case-by-case basis and require written approval from the PI, in accordance with institutional and ethical guidelines. Data sharing is not applicable at this stage as this manuscript describes a study protocol. After study completion, de-identified data may be made available upon reasonable request to the corresponding author and with appropriate ethical approvals.
