# Supplementary material for: Church-based problem-solving therapy for adolescent girls and young women with a history of gender-based violence in Zambia: study protocol for a hybrid type 1 randomized controlled trial
Source: Trials. 2026 Apr 10;27:370. doi: 10.1186/s13063-026-09625-3 (PMC13174015; doi:10.1186/s13063-026-09625-3)
Supplement: Supplementary file 1 — Additional file 1. Label as WHO Trial Registration Data. [file 13063_2026_9625_MOESM1_ESM.docx]

*WHO Trial Registration Data*

| Data Category | Information |
| --- | --- |
| Primary registry and trial identifying number | ClincalTrials.gov  NCT07132905 |
| Date of registration in primary registry | 20 August 2025 |
| Secondary identifying numbers | N/A |
| Source(s) of monetary or material support | UCLA-Charles R. Drew University Center for AIDS Research (CFAR) grant AI152501  University of California, San Francisco |
| Primary sponsor | UCLA-Charles R. Drew University Center for AIDS Research (CFAR) grant AI152501 |
| Secondary sponsor(s) | University of California, San Francisco |
| Contact for public queries | Moomba M Thornicroft, MPH  [mcpals4life@gmail.com](mailto:mcpals4life@gmail.com?subject=NCT07132905,%20IRB-23-1685-AM-005,%20Mpata%20Yathu%20Trial%20for%20Young%20Women%20in%20Zambia) |
| Contact for scientific queries | Charisse V Ahmed, PhD  [charisse.ahmed@ucsf.edu](mailto:charisse.ahmed@ucsf.edu?subject=NCT07132905,%20IRB-23-1685-AM-005,%20Mpata%20Yathu%20Trial%20for%20Young%20Women%20in%20Zambia) |
| Public title | Mpata Yathu Trial for Young Women in Zambia (MYT) |
| Scientific title | Church-Based Problem-Solving Therapy for Adolescent Girls and Young Women with a History of Gender-Based Violence in Zambia: Study Protocol for a Hybrid Type 1 Randomized Controlled Trial |
| Health condition(s) or problem(s) studied | HIV, Common Mental Health Problems, PTSD - Post Traumatic Stress Disorder, Depression Disorder, Anxiety |
| Intervention(s) | Problem-Solving Therapy |
| Key inclusion and exclusion criteria | Inclusion Criteria   1. aged 15 to 24 years; 2. Speak Nyanja, Bemba, and/or English fluently; 3. Reside in the Matero or Chawama constituency area during the time of recruitment; 4. Report lifetime exposure to GBV, as assessed by the WHO Multi-country Study on Women's Health and Domestic Violence Against Women instrument. 5. Exhibit moderate depressive symptoms indicated by a score of 10-14 on the 9-item Patient Health Questionnaire (PHQ-9), or common mental disorder (CMD) symptoms (e.g., depression, anxiety) as indicated by a score of 9 or higher on the 14-item Shona Symptom Questionnaire (SSQ-14); and 6. Be living with HIV or demonstrate HIV risk behaviors, as defined by validated items from the World AIDS Foundation survey, including unprotected sex, multiple sexual partners, coerced sex, or transactional sex.   Exclusion Criteria   1. Require emergency treatment for any crisis (mental, physical, emotional) at the time of screening 2. Report severe symptoms of depression (score > 14 on PHQ-9) or no to mild depression symptoms (PHQ-9 score <10), and/or severe anxiety symptoms using the Generalized Anxiety Disorder 7-item scale or GAD-7 (score >14 on GAD-7) 3. Have intellectual or cognitive disabilities that limit their ability to complete the screening tools, interact with a lay counselor and/or provide informed consent; and/or 4. Are considered in immediate danger (e.g., reoccurring physical violence) during time of the study. 5. Are currently receiving formal mental health counseling or psychotherapy (to avoid duplication of care and potential confounding effects). |
| Study type | Interventional  Allocation: Randomized  Interventional Model: Parrell Assignment  Masking: Single |
| Date of first enrolment | October 2025 (anticipated) |
| Target sample size | 180 (90 per arm) |
| Recruitment status | Recruitment will start October 2025 |
| Primary outcome(s) | Change in symptoms of common mental disorders (CMDs) as measured by the Shona Symptom Questionnaire (SSQ-14) |
| Key secondary outcomes | - Change in depressive symptoms as measured by the Patient Health Questionnaire-9 (PHQ-9) - Change in anxiety symptoms as measured by the Generalized Anxiety Disorder-7 (GAD-7) scale - Change in PTSD symptoms as measured by the Child PTSD Symptom Scale (CPSS) |
