# Supplementary material for: Church-based problem-solving therapy for adolescent girls and young women with a history of gender-based violence in Zambia: study protocol for a hybrid type 1 randomized controlled trial
Source: Trials. 2026 Apr 10;27:370. doi: 10.1186/s13063-026-09625-3 (PMC13174015; doi:10.1186/s13063-026-09625-3)
Supplement: Supplementary file 2 — Additional file 2. Label as informed consent form template. [file 13063_2026_9625_MOESM2_ESM.docx]

**Informed Adult Consent and Minor Assent: Mpata Yathu Pilot Study**

**CONSENT TO PARTICIPATE IN RESEARCH**

**INTRODUCTION**

Dr. Charisse Ahmed, PhD, RN, from the University of California, San Francisco (UCSF), Dr. Kathryn Dovel PhD, MPH from the University of California, Los Angeles (UCLA), and Dr. Noé Rubén Chávez, PhD from Charles R. Drew University of Medicine & Science are conducting a research study. Dr. Charisse Ahmed is considered the Principal Investigator for this study. A Principal Investigator (or PI) is the person in charge of a research project. They lead and oversee the study, making important decisions and ensuring everything runs smoothly. They are often the main point of contact for the project and are responsible for its success. You are being invited to take part in a research study. This form tells you what this research study is about. It also explains what will happen if you decide to be in this study and any risks of taking part. If there is anything on this form that you do not understand, please ask questions.

**What is a research study?** A research study collects information about something that people don’t understand well. The study helps us to learn more. By learning more, we can find new ways to help people.

**Why am I being asked to be in this study?** This study is for young women ages 15–24 who may have gone through difficult life events and are looking for emotional support. You are being asked to be in this research study because you are a young woman 15 to 24 years old who may be experiencing stress, sadness, or emotional difficulties due to past difficult life experiences. If you decide not to be in the study your care will not be changed because of that decision. The choice to be in the study is totally up to you. You do not require permission from your parent or guardian to participate in this study.

**What is the purpose of this research study?** We would like to understand how to help young women who may be experiencing stress, sadness, or emotional difficulties due to past difficult life experiences. We want to understand whether providing counseling sessions in churches can help young people feel more supported and make it easier to take care of their health and wellbeing. The study will do this by inviting young women to the Mpata Yathu intervention, where they will meet trained lay counselors. The lay counselors are community members who are not professional therapists but have been trained to support people in managing life challenges and finding solutions. Mpata Yathu uses problem-solving therapy. Problem-solving therapy is a treatment that helps people take action in their lives, helping them cope with difficulties, and teaching them to proactively solve their problems. Problem-solving therapy helps people directly work on life's challenges. Problem-solving therapy can help with achieving goals, finding purpose, reducing depression, managing anxiety, and solving relationship problems. Problem-solving therapy has been researched and shown to be helpful for all ages.

**What will happen during the study?**

- After you complete the eligibility screening and are determined to be eligible for this study, you will be enrolled in the study and asked to complete the same questionnaire at 3 different times—when you first enroll in the study, three months after you enroll, and 6 months after you enroll. The questionnaire contains different surveys that will help us evaluate your experiences and how you are feeling, including how you’ve been feeling emotionally, such as feeling down, stressed, or worried; how you are managing daily challenges; how you take care of your health; and how safe you feel in your surroundings. We will also look at whether changes in how you’re feeling emotionally may be connected to how you take care of your health, including HIV prevention or treatment. A member of our study team will ask you the questions from the questionnaire and record your responses. The questionnaire will take about 1 hour to complete.
- Once you are enrolled in the study, you will be randomized to a certain group. Randomization means that you are assigned to a group by chance (like a flip of a coin). It is used to make sure study results are not influenced by the selection of participants in one group as compared to another. A computer program will place you in one of the groups. Neither you nor the researchers can choose the group you will be in. You will have an equal chance of being placed in any group. You will be randomized into one of the study groups described below.
  - If you are in Group 1: Immediately after you complete your first questionnaire, you will receive the weekly counseling sessions delivered by a lay counselor once per week for up to 6 weeks.
  - If you are in Group 2: Three months after you complete your first questionnaire, you will receive the weekly counseling sessions delivered by a lay counselor once per week for up to 6 weeks. You will be contacted by a member of our study team when it is time for you to start your counseling sessions.
- You will have a total of 6 counseling sessions with a trained lay counselor. Each session will last about 1 hour. You will attend 1 session per week at a private space at [name church]. Your counseling sessions will be audio recorded, but only if you give permission. These recordings help us make sure the counselors are offering support in the best way possible. During the first two weeks of the study, we will review all recordings (if you agree to be recorded) so that we can give helpful feedback to the counselors. After that, we will review a small number of sessions, which means about one recording for every five participants in the study. You can say no to being recorded at any time. You may also use a different name during your sessions to protect your privacy.
- Once you have completed the counseling sessions, you may be selected to be interviewed by a member of our study team. We will ask you questions about your thoughts, emotions, and how helpful the counseling sessions were for you. The interview session will last about 1 hour. We will audio record the interview so that we can have a record of it for purposes of improving knowledge on how to best deliver mental health interventions for young women like you.

**What are the risks of taking part in this research study?** There is a small chance you may feel uncomfortable or experience some emotional distress when answering some of the questions on the questionnaires or during the activities of the study. If you are not comfortable with answering any of the questions, you can choose not to answer.

**If I take part in this research, how will my privacy be protected? What happens to the information you collect?** Any information you give us will be de-identified which means we will remove your name and instead use a subject number. We will use the information you give us, but it will be linked to your subject number instead of your name. Also, each lay counselor is required to sign a confidentiality agreement to ensure protection of your privacy. However, if you disclose thoughts of harming yourself or someone else or tell your counselor that you are currently in an unsafe situation, the lay counselors and the rest of our study team are required to report this so that you can receive the help you need. If you agree to be recorded, the audio recordings of your interviews or counseling sessions will only be accessed by the study team. These recordings will be destroyed two years after the study ends.

We will publish the results of this study so others can learn from what we find. This may include your answers from assessments such as surveys, interviews, or other questions we ask you. We may also share overall numbers, such as how many people stayed in the study, how many stopped participating, and how many were connected to support services. In our published reports, we will never include your name, phone number, or any personal information that could identify you. Your responses will always be kept private and will not be linked to you.

Your information in this research will be collected, stored, and managed using a secure web application called REDCap. Only the study team, which includes the study’s main researcher or Principal Investigator (Dr. Charisse Ahmed) and local study staff, will have access to this data. Since this research is funded by the U.S. National Institutes of Health, the information gathered may be reviewed by them or other U.S. government agencies. The information you provide during this study will be de-identified which means all personal details like your name, address, and phone number will be replaced with a code number. Other researchers can request access to this de-identified data for their own research purposes by Dr. Charisse Ahmed. Dr. Charisse Ahmed will carefully assess each request to protect your privacy. Sharing this data with other researchers helps us learn more quickly about effective treatments. If you prefer not to share your data with other researchers, you can get in touch with the study coordinator or Dr. Charisse Ahmed. Your de-identified data may be kept for use in future research. But we will make sure to protect the privacy of your information.

This research is covered by a Certificate of Confidentiality from the United States National Institutes of Health. This provides extra protections to the data when they are maintained in the United States. This means that the researchers cannot release or use information, documents, or samples that may identify you in any action or suit unless you say it is okay. They also cannot provide them as evidence unless you have agreed.  This protection includes federal, state, or local civil, criminal, administrative, legislative, or other proceedings. There are some important things that you need to know. The Certificate DOES NOT stop reporting that local laws require. Some examples are laws that require reporting of child or elder abuse, some communicable diseases, and threats to harm yourself or others. The Certificate CANNOT BE USED to stop a sponsoring United States federal or state government agency from checking records or evaluating programs. The Certificate also DOES NOT prevent your information from being used for other research if allowed by federal regulations in Zambia and the United States. Researchers may release information about you when you say it is okay. For example, you may give them permission to release information to medical providers or any other persons not connected with the research. The Certificate of Confidentiality does not stop you from willingly releasing information about your involvement in this research. It also does not prevent you from having access to your own information.

**Are there any benefits to being in the study?** Your participation in this intervention may help improve your mood and stress levels, although this is not guaranteed. Lastly, the information gathered in this study will be used to help improve services available to young women with stress and worry in Zambia.

**Will I be paid to participate in the study?** You will receive a transportation reimbursement amount of 50 Zambian Kwacha each time you attend a counseling session and for each questionnaire you complete (at the three time points listed above).

**What happens if I decide not to take part in the study?** Nothing will happen to you if you decide not to participate in this study.

**If I have any questions, concerns, or complaints about this research study, who can I talk to?** You may contact the following individuals for assistance or information about this study:

- **Study Coordinator:** Responsible for overseeing the research activities. [Insert contact information here]. They can be reached directly at this number or on WhatsApp.
- **Mental Health Specialist:** Provides support for emotional and mental health concerns related to the study. [Insert contact information here]. They can be reached directly at this number or on WhatsApp.
- **Church Staff:** Church staff can assist with study-related concerns and can be reached in person during [insert available hours here] or contacted directly at [insert contact information here] or via WhatsApp.
- **School Staff:** School staff at [name school] are available during [insert available hours here] for in-person discussions or can be contacted directly at [insert contact information here].

**If you feel like harming yourself at any point during the study, please contact our mental health specialist immediately at [insert number here], available directly or via WhatsApp. If you cannot reach them, call Zambia’s emergency number at 999. You can also inform your lay counselor, the study coordinator, or [name contact person(s) at the church] for assistance. If the study team determines that your health or safety is at risk, a licensed clinician on our team will evaluate you and refer you to additional services as needed for your safety and mental wellbeing.**

**Our team will also check for any signs that you may be feeling unsafe or at risk during the study. If we think you may be in danger or need more support, we will follow up and keep a private record to help make sure you get the help you need.**

**If you tell your counselor that you are currently experiencing harm or unsafe treatment from someone, we are required by law to report this to appropriate authorities, which may include the police. Our study team will support you during this process and help connect you to services that can ensure your safety.**

The lead of this research study is Dr. Charisse Ahmed. Dr. Ahmed can also be contacted directly via email at charisse.ahmed@ucsf.edu or via WhatsApp (+1 352-222-0929).

If you have questions about your rights as a research participant, or you have concerns or suggestions and you want to talk to someone other than the researchers, you can contact The University of Zambia Biomedical Research Ethics Committee (UNZAREC) by phone: +260-1-256067; by email: [unzarec@unza.zm](mailto:unzarec@unza.zm) or by mail: P.O. Box 50110, Lusaka, Zambia. You may also contact the UCLA Office of the Human Research Protection Program by phone: +1 (310) 206-2040; by email: [participants@research.ucla.edu](mailto:participants@research.ucla.edu) or by mail: Box 951406, Los Angeles, CA 90095-1406.

**What are my rights if I take part in this study?**

- You can choose whether or not you want to be in this study, and you may withdraw your consent and discontinue participation at any time.
- No matter what choice you make, you won't be punished, and you won't lose any benefits you were supposed to receive (such as referral to a provider for your mental wellness).
- You may refuse to answer any questions that you do not want to answer and remain in the study.
- You have the right to review any information we collect from you, including audio recordings, to determine whether they should be edited or erased in whole or in part.

***You will be given a copy of this information to keep for your records.***

**How do I give my agreement to participate?** By providing your signature and the date below, that will tell the PI and the research team that you have agreed to participate in this study. Please ask any questions you have about this study now. If you decide not to take part at any time during the study, you can always change your mind and tell the research team that you wish to stop taking part in the study. If you understand this study and want to take part, we ask that you sign below. By signing, you agree that you have had your questions answered and that you are willing to take part in the study.

You have read or heard the consent form read to you and you understand the consent form. You have been given the chance to ask questions and have had them answered to your satisfaction. You agree to take part in this research study. Upon signing below, you will receive a copy of the consent form.

Name of study participant: _______________________________________________________

|  |  |  |
| --- | --- | --- |
| Name of Person Consenting | Date/Time |  |
| Signature (or thumb print) of Person Consenting | Date/Time |  |

|  |  |  |  |  |
| --- | --- | --- | --- | --- |
| Name of Witness to Consent |  | Signature of Witness to Consent |  | Date/Time |

| ----------------------------------------------------------------------------------------------------------------------------  Assent certification (if person consenting is less than 18 years of age):  I have explained the study and the procedures involved to  _____________________________________ in terms he/she could understand and that he/she freely assented to take part in the study. | | | |
| --- | --- | --- | --- |
| Signature of Person Obtaining Assent |  |  | Date/Time |

|  |  |
| --- | --- |
| Name of Minor Assenting | Date/Time |
| Signature (or thumb print) of Minor Assenting | Date/Time |
